# The immediate effects of the severe acute respiratory syndrome (SARS) epidemic on childbirth in Taiwan

**DOI:** 10.1186/1471-2458-5-30

**Published:** 2005-04-04

**Authors:** Cheng-Hua Lee, Nicole Huang, Hong-Jen Chang, Yea-Jen Hsu, Mei-Chu Wang, Yiing-Jenq Chou

**Affiliations:** 1 Institute of Health Care and Hospital Administration, National Yang Ming University, 155 Li-Nong Street, Section 2, Taipei, 112 Taiwan; 2 Bureau of National Health Insurance, Taipei, Taiwan; 3 Department of Health Education, National Taiwan Normal University, Taipei, Taiwan; 4 Institute of Health and Welfare Policy, National Yang Ming University, Taipei, Taiwan; 5 Department of Social Medicine, National Yang Ming University, Taipei, Taiwan

## Abstract

**Background:**

When an emerging infectious disease like severe acute respiratory syndrome (SARS) strikes suddenly, many wonder the public's overwhelming fears of SARS may deterred patients from seeking routine care from hospitals and/or interrupt patient's continuity of care. In this study, we sought to estimate the influence of pregnant women's fears of severe acute respiratory syndrome (SARS) on their choice of provider, mode of childbirth, and length of stay (LOS) for the delivery during and after the SARS epidemic in Taiwan.

**Methods:**

The National Health Insurance data from January 01, 2002 to December 31, 2003 were used. A population-based descriptive analysis was conducted to assess the changes in volume, market share, cesarean rate, and average LOS for each of the 4 provider levels, before, during and after the SARS epidemic.

**Results:**

Compared to the pre-SARS period, medical centers and regional hospitals dropped 5.2% and 4.1% in market share for childbirth services during the peak SARS period, while district hospitals and clinics increased 2.1% and 7.1%, respectively. For changes in cesarean rates, only a significantly larger increase was observed in medical centers (2.2%) during the peak SARS period. In terms of LOS, significant reductions in average LOS were observed in all hospital levels except for clinics. Average LOS was shortened by 0.21 days in medical centers (5.6%), 0.21 days in regional hospitals (5.8%), and 0.13 days in district hospitals (3.8%).

**Conclusion:**

The large amount of patients shifting from the maternity wards of more advanced hospitals to those of less advanced hospitals, coupled with the substantial reduction in their length of maternity stay due to their fears of SARS could also lead to serious concerns for quality of care, especially regarding a patient's accessibility to quality providers and continuity of care.

## Background

The first probable case of severe acute respiratory syndrome (SARS) in Taiwan was identified on March 14, 2003 [1]. The epidemic became elevated at the end of April and reached its peak in May and June. Finally, the epidemic ended when Taiwan was officially removed from the World Health Organization (WHO) list of SARS affected countries on July 5, 2003 [1-3]. The fear of SARS spread over all of Taiwan and was mainly directed at those regional hospitals and medical centers where outbreaks had occurred and where SARS patients were being treated. People's fears originated with the novelty of the disease, its rapid nosocomial transmission, and the apparent vulnerability of hospitals and health care workers. Many people started to wonder whether the overwhelming fears of SARS directed at the regional hospitals and medical centers changed a patient's care seeking behavior and even physicians' treatment patterns, and thereby had possible health consequences, especially for those whose survival or state of health depended on routine and/or continuous medical care.

Delivery of a child is a classic example. Under normal circumstances, distance and quality are believed to be two major determinants in a patient's choice for obstetrics care [4-7]. In this study, we sought to estimate the influence of people's fears of SARS on people's choice of provider, mode of child delivery, and length of hospital stay before, during and after the delivery. The people's fears include fears of patients and fears of doctors as choice of mode of delivery and length of stay tend to be a joint decision. The fears of doctors to SARS may also reduce cesarean section rate and shorten the length of stay for lowering the possibility of acquiring SARS. Considering the fact that hospital levels [5,8-15], (mode of delivery [16,17], and length of maternity stay [18-25] are significantly associated with postpartum maternal and neo-natal health status, the question whether the fears of SARS led patients to transfer from a more advanced hospital to a less advanced hospital becomes an important quality of care concern. Health consequences resulting from these behavioral changes due to people's fears of SARS should not be overlooked [26-29]. The results could provide public health agencies an important reference when assessing the consequences of the SARS epidemic on quality of care. So that when SARS re-emerges or other similar new infectious disease emerges, it can guide obstetricians and public health professionals to prevent avoidable health consequences because of people's fears concerning these new and strongly infectious diseases.

## Methods

### Obstetrics services under the NHI program

Since its implementation in March 1995, the National Health Insurance (NHI) program provides a mandatory and comprehensive universal health care coverage for all Taiwanese residents. For obstetrics care, a wide range of services including pre-natal, delivery, and neo-natal care are extensively covered under the NHI program. Both vaginal and cesarean deliveries are covered and no co-insurance is required for child delivery. The program offers patients complete freedom of choice among providers and methods of delivery. In order to contain the in-patient costs for Western medical services, the NHI program has instituted the case-payment method for 50 clearly defined medical conditions, and they include both vaginal delivery and cesarean delivery. Case payment is similar to the DRG (diagnosis-related groups) payment system in the U.S. It bundles itemized medical services essential for each condition and provides financial incentives for a more efficient delivery process. The case payment system reimburses the provider on a per-case basis, and the payment varies by accreditation level of the provider. It also assumes that the complexity or quality of care also varies by provider level. The current accreditation system categorizes medical institutions into 4 levels, from most advanced to most basic: medical center, regional hospital, district hospital, and clinic. The accreditation criteria include infrastructure, capacity, manpower, volume, management and administrative processes. For vaginal delivery, the case payment ranges from NT$17,420 per case in medical centers to NT$15,100 per case in clinics. For cesarean delivery, the range is larger (from NT$32,330 per case in medical centers to NT$27,170 per case in clinics). Furthermore, the National Health Insurance Law prohibits its contracted providers from any direct/extra billing for any service covered by the NHI program.

### Study design and statistical analyses

This study conducted a population-based descriptive analysis of changes in market share, cesarean rate, and length of maternity stay, for each of the four levels of provider, before, during and after the SARS epidemic. This study retrieved all 448,365 NHI in-patient claims for childbirth from January 01, 2002 to December 31, 2003. Vaginal deliveries were those coded with the NHI Case Payment-DRG 0373A and 0373C. Cesarean deliveries were those coded with the NHI Case Payment-DRG 0371A and 0373B. Each claim cites the patient, provider, diagnoses, treatment procedures, mode of delivery, and admission and discharge dates. A unique provider identification number was used to link the NHI provider file, which identifies provider accreditation level.

As the SARS epidemic started in March, elevated in May, and finally ended in July, we divided the study period into 5 sub-periods: pre-SARS period from January 2002 to February 2003 (t_0_), initial SARS period from March 2003 to April 2003 (t_1_), peak SARS period from May 2003 to June 2003 (t_2_), final SARS period from July 2003 to August 2003 (t_3_) and post-SARS period from September 2003 to December 2003 (t_4_). Monthly average estimates for market share, cesarean rate, length of maternity stay, all by accreditation level were calculated and expressed in numbers and percentages. Mean differences in average cesarean rate and average length of maternity stay between the pre-SARS and the peak periods (t_2 _- t_0_), and between the pre-SARS and post-SARS periods (t_4 _- t_0_) were determined by 2-tailed t tests. The data were managed and analyzed by using SAS, Version 8.2. All analyses were tested for significance by using an alpha of .05. Since no human participants were involved, no IRB approval is necessary. The confidentiality assurances were addressed by abiding the data regulations of the Bureau of National Health Insurance, which stipulated the data only for use of this research.

## Results

### Changes in market share

**Figure 1:**
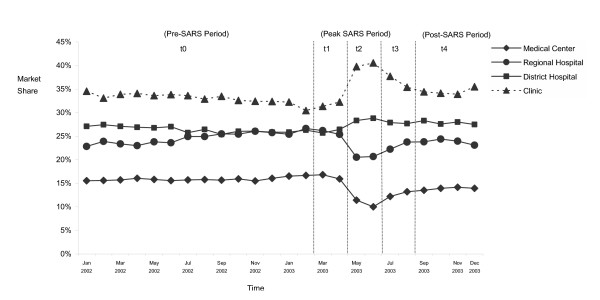
Trends in market shares of childbirth service in Taiwan by provider's accreditation level, January 2002–December 2003.

Figure 1 shows the changes in the market share of delivery services by accreditation level of provider in the five sub-periods. In terms of market share, before the SARS epidemic, although clinics still had the largest market share, they were slowly losing their share to their powerful competitors, the medical centers and the regional hospitals. However, people's fears of SARS reversed this situation during the SARS epidemic, as patients switched to clinics during the SARS epidemic to deliver their child, rather than in the larger academic medical centers and regional hospitals. A dramatic drop in market share was observed for both the medical centers and the regional hospitals during the SARS epidemic. While at the same time both district hospitals and clinics gained market share. In addition, Table 1 shows the changes in monthly average number of deliveries, and the market share of delivery services by accreditation level of provider in the 3 key sub-periods (pre-SARS, peak-SARS, and post-SARS periods). Generally speaking, over the past several years Taiwan has experienced a constant decreasing fertility rate. On average, there were 2,189 fewer deliveries each month during the peak SARS period than during the pre-SARS period. Compared to the pre-SARS period (t_0_), medical centers and regional hospitals dropped 5.2% and 4.1% in market shared during the peak SARS period (t_2_), but district hospitals and clinics increased 2.1% and 7.1%, respectively. Although medical centers and regional hospitals regained some of their lost market share during the post-SARS period (t_4_), they still did not fully regain their market shares at the pre-SARS level (medical centers: 2.0%; regional hospitals: 0.8%).

**Table 1:** Average Volumes of Childbirth Service in Taiwan by Provider's Accreditation Level and Time Period, January 2002–December 2003.

	**t_0_**		**t_2_**		**t_4_**		**t2-t0**		**t4-t0**	
					
	**Pre-SARS Period (Jan 2002~Feb 2003)**		**Peak SARS Period (May~Jun 2003)**		**Post-SARS Period (Sep~Dec 2003)**		**Difference**		**Difference**	
					
**Accreditation Level**	**No.**	**%**	**No.**	**%**	**No.**	**%**	**No.**	**%**	**No.**	**%**
					
**Medical Center**	3,044	15.9	1,820	10.7	2,574	13.9	-1,225	-5.2	-471	-2.0
**Regional Hospital**	4,731	24.7	3,501	20.6	4,415	23.8	-1,230	-4.1	-316	-0.8
**District Hospital**	5,067	26.4	4,853	28.5	5,156	27.8	-214	2.1	90	1.4
**Clinic**	6,346	33.1	6,826	40.2	6,382	34.4	480	7.1	36	1.4
					
**Total**	19,188	100.0	16,999	100.0	18,527	100.0	-2,189		-661	

### Changes in cesarean rate and length of maternity stay

**Figure 2:**
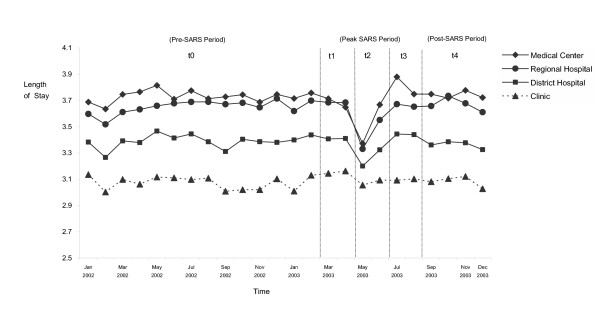
Trends in length of maternity stay in Taiwan by provider's accreditation level, January 2002–December 2003.

Table 2 compares the changes in monthly average cesarean section rates and length of stay (LOS) in the pre-, peak- and post-SARS periods (t_0_, t_2_, and t_4_). Compared to the pre-SARS period, we observed only a marginal decrease in overall cesarean rate (1.0%), but a significantly larger increase in cesarean rate in medical centers (2.2%) and significantly decrease in clinics (1.9%) during the peak SARS period (t_2_). One plausible explanation is that as normal or less complicated deliveries shifted to lower level hospitals or clinics, those cases that remained in the medical centers tended to be complicated ones which required cesarean sections. Hence, although women shifted their place of delivery from higher level hospitals to lower level hospitals/clinics due to a greater risk of exposure to SARS at these higher level hospitals, their choice of delivery method did not seem to change. In addition, the average cesarean section rate in medical centers returned to the pre-SARS level during the post-SARS period while the cesarean section rates in lower level hospitals and clinics showed the opposite. The cesarean section rates in regional, district hospitals and clinics dropped significantly during the post-SARS period from their levels before the SARS epidemic. Furthermore, Table 2 and Figure 2 present changes in average LOS among different provider levels. As expected, in order to reduce their potential risk of exposure to SARS, people tried to minimize their maternal stay in a hospital as much as possible. The overall average length of stay decreased from 3.40 days to 3.25 days from the pre-SARS period (t_0_) to the peak SARS period (t_2_), and then returned to 3.39 days after the SARS epidemic (t_4_). More specifically, significant reductions in average LOS were observed in all hospital levels except for clinics at t_2_. Average length of stay was shortened by 0.21 days in medical centers (5.6%), 0.21 days in regional hospitals (5.8%), and 0.13 days in district hospitals (3.8%). Average LOS in clinics remained basically unchanged. However, as soon as the SARS epidemic ended, average LOS in most hospitals and clinics not only returned to the pre-SARS level, but became slightly longer than it was prior to SARS, which is not significant statistically speaking.

**Table 2:** Average Cesarean Section Rates and Length of Maternity Stay in Taiwan by Provider's Accreditation Level and Time Period, January 2002–December 2003.

	**t_0_**	**t_2_**	**t_4_**	**t2-t0**	**t4-t0**
					
	**Pre-SARS Period (Jan 2002~Feb 2003)**	**Peak SARS Period (May~Jun 2003)**	**Post-SARS Period (Sep~Dec 2003)**	**Difference**	**Difference**
**C-Section Rate**	**%**	**%**	**%**	**% (S.E)**	**% (S.E)**
					
**Medical Center**	36.1	38.3	36.1	2.2 (0.7) *	0.0 (0.6)
**Regional Hospital**	32.8	32.3	31.7	-0.5 (0.6)	-1.1 (0.5) *
**District Hospital**	34.3	33.2	30.5	-1.1 (0.5) *	-3.7 (0.4) **
**Clinic**	34.5	32.6	31.3	-1.9 (0.5) **	-3.2 (0.4) **
					
**Total**	34.3	33.3	31.9	-1.0 (0.4) *	-2.4 (0.3) **

**Average LOS**	**Day**	**Day**	**Day**	**Day (S.E)**	**Day (S.E)**
					
**Medical Center**	3.73	3.52	3.74	-0.21 (0.05) **	0.01 (0.02)
**Regional Hospital**	3.65	3.44	3.67	-0.21 (0.05) **	0.02 (0.03)
**District Hospital**	3.39	3.26	3.36	-0.13 (0.04) **	-0.03 (0.03)
**Clinic**	3.07	3.07	3.08	0.00 (0.04)	0.01 (0.03)
					
**Total**	3.40	3.25	3.39	-0.15 (0.04) **	-0.01 (0.02)

*P < 0.05, two-tailed test; **P < 0.01, two-tailed test

## Discussion

Our results, based on a population-based study, demonstrate that if the use of medical care required was essential and could not be deferred, such as childbirth, the fears of SARS, including both fears of patients and fears of doctors, had a significant influence on patients' preference for provider and on their length of in-patient stay. However, it did not necessarily influence their choice of therapies during the SARS epidemic. Even though the SARS influence upon a patient's decision regarding the length of stay ended with the end of the epidemic, the fear of SARS remained influential on how people chose their place of delivery. During the SARS epidemic, large amounts of patients shifting from more advanced hospitals to less advanced hospitals and substantially, reducing their length of maternity stay simply out of fear could result in a serious concern for quality of care, especially for a patient's accessibility to quality care and the continuity of that care. For example, patients transferring to a less advanced hospital could severely compromise their access to more sophisticated technologies and interrupt their continuity of care. This in turn could endanger the lives of the mothers and their babies [5,8-14]. (Significantly shorter length of stay increases the risk of a premature discharge and compromise the quality of care, which again could lead to adverse health outcomes for both mother and baby [23-25,30].

Furthermore, not only the physicians who may directly encounter with SARS patients need to be alert and better trained for this type of outbreak, obstetricians should also be aware and extremely cautious about suddenly substantial patient shift and strong demand of shorter length of maternity stay posed by the public's fear of such an outbreak. As these sudden changes in people's behaviors are likely to hinder patient's continuity of care and cause adverse maternal and neonatal health consequences, it is essential for obstetricians to be well prepared to deal with these consequences during an outbreak.

A few study limitations should be noted. First, due to data and time constraints, this study only shows the immediate impacts of the fears of SARS on the shifting of patients from one facility to another, as well as their length of stay. This study cannot confirm whether these changes led to any long term adverse maternity or perinatal outcomes. If in the post-SARS period, mother's and child's morbidity and mortality remained the same, the residual increase of child births in clinics and district hospitals would be a positive impact since it reduces health care costs without compromising health outcomes. Future research with a longer post-SARS observational period and more detailed maternal and perinatal outcome information could help to advance our knowledge in this regard. Second, the significant amount of patient shifting observed among provider levels suggests that the fear of SARS changed people's preference as to their choice of provider during the SARS epidemic. Whether this influence will persist remains to be seen. Third, since we only focused on childbirth in this study, the results may not be generalizable to other medical conditions. Patient behavior may very well differ for medical conditions/diseases with different levels of severity and/or medical urgency. Furthermore, the influence of the fear of SARS on other important patient behaviors, such as their decision to seek care or not during the SARS epidemic if their medical needs could be deferred or suspended to a later time, remains uncertain. There are many questions that remain to be answered concerning the possible impacts of SARS on various aspects of health care.

## Conclusion

In terms of policy implications, the medical centers and regional hospitals in Taiwan took on the majority of the responsibility in caring for the more severe SARS cases, and by doing so lost their market share to district hospitals and clinics because of people's fear of SARS. The BNHI negotiated with these hospitals to compensate them for their loss of revenue during the SARS epidemic, and bring it up to the previous year's level. It is expected that by minimizing a hospital's financial loss it will increase that hospitals' willingness to admit SARS patients and secure people's access to proper medical care in the likelihood of a re-emergence of a SARS epidemic or any other pandemic. Strategies which help to restore people's confidence in those hospitals that have admitted SARS patients should be part of the long term solution.

Finally, while internationally all of the global attention is focused on the direct causalities of SARS, serious quality of care concerns resulting from people's behavioral changes due to their fears of SARS should not be overlooked. Taiwan's experience could provide valuable lessons to other countries in assessing full impacts of the SARS epidemic and help to minimize adverse health consequences when SARS or other similar pandemic emerge.

## List of abbreviations

SARS: severe acute respiratory syndrome

NHI: the National Health Insurance

LOS: length of stay

C-section: cesarean section

No: number

Jan: January

Feb: February

Mar: March

Apr: April

Jun: June

Jul: July

Aug: August

Sep: September

Oct: October

Nov: November

Dec: December

## Competing interests

The author(s) declare that they have no competing interests.

## Authors' contributions

CHL planned the study and supervised all aspects of its implementation. NH assisted with the study and led the writing. HJC synthesized analyses and contributed to the writing of the article. YJH and YJC contributed to the design, analyzed the data, commented on the interpretation of the results. MCW assisted with the study and completed the statistical analyses. All authors helped to conceptualize ideas, interpret findings, and review drafts of the manuscript. All authors read and approved the final manuscript.

## Pre-publication history

The pre-publication history for this paper can be accessed here:


